# Gastric Regional Lymph Node Metastases from a Squamous Cell Carcinoma of Unknown Primary Site: A Case Report

**DOI:** 10.70352/scrj.cr.25-0727

**Published:** 2026-02-10

**Authors:** Toshiaki Komo, Yoichi Sugiyama, Takaaki Suwa, Ryohei Watanabe, Masayuki Mori, Yoshifumi Kondo, Tetsuhiro Hara, Takuro Yamaguchi, Tatsuya Tazaki, Mohei Koyama, Atsushi Nakamitsu, Shinya Takahashi, Masaru Sasaki

**Affiliations:** 1 Department of Surgery, JA Hiroshima General Hospital, Hatsukaichi, Hiroshima, Japan; 2 Department of Surgery, Graduate School of Biomedical and Health Sciences, Hiroshima University, Hiroshima, Hiroshima, Japan

**Keywords:** cancer of unknown primary site, squamous cell carcinoma, lymph node metastasis, gastric submucosal tumor

## Abstract

**INTRODUCTION:**

A cancer of unknown primary site is a malignant tumor for which the primary site is unknown despite a thorough examination, and which has been histologically proven to be a metastatic lesion. Metastases to intraperitoneal and to gastric regional lymph nodes are rare.

**CASE PRESENTATION:**

A 75-year-old woman was diagnosed with a gastric submucosal tumor with infiltration to other organs. Endoscopic ultrasound-guided fine needle aspiration revealed cells that appeared to be derived from epithelial tissue, but a definitive diagnosis could not be obtained. Because the possibility of gastric cancer could not be ruled out, an open proximal gastrectomy with systematic lymph node dissection and combined resection of other organs were performed. A grade II pancreatic fistula developed, but resolved with conservative treatment, and the patient was discharged 15 days after surgery. Histopathologically, the tumor was a lymph node metastasis consisting of squamous cell carcinoma cells that had grown primarily outside the gastric wall, but involved the gastric wall and pancreas and protruded into the gastric mucosa. Thirty-five gastric lymph nodes were dissected, and metastases were found in five of them. Primary squamous cell carcinoma of the stomach and pancreas was ruled out. Because no head and neck, esophageal, or pulmonary lesions that could be squamous cell carcinoma were identified, the primary tumor could not be identified. The diagnosis was a gastric regional lymph node metastasis of a cancer of unknown primary site protruding into the gastric wall. Nivolumab was initiated after surgery, and the patient has remained alive and free of recurrence 7 months after surgery.

**CONCLUSIONS:**

In cases of metastases originating from a cancer of unknown primary site to lymph nodes in the gastric region, the removal of the affected lymph nodes followed by inability to detect the primary lesion might be considered to be equivalent to an R0 resection.

## Abbreviations

CUP
: cancer of unknown primary site
SUV
: standardized uptake value

## INTRODUCTION

A CUP is a malignant tumor for which the primary site is unknown despite a thorough examination, and which has histological characteristics of a metastatic lesion.^1)^ It accounts for 2%–4% of all diagnosed cancers.^2)^ The majority of these are adenocarcinomas, with squamous cell carcinomas accounting for 5% of diagnosed cancers.^3)^

Approximately 10%–40% of metastatic lesions in patients with CUP are metastatic lymph nodes.^1)^ However, metastases to intraperitoneal lymph nodes are uncommon^4)^ and metastases to gastric regional lymph nodes are rare.

Herein, we report a case of gastric regional lymph node metastases from a squamous cell carcinoma of unknown primary site that was discovered to be a gastric submucosal tumor with invasion of other organs, and which was resected by a gastrectomy.

## CASE PRESENTATION

**Fig. 1:**
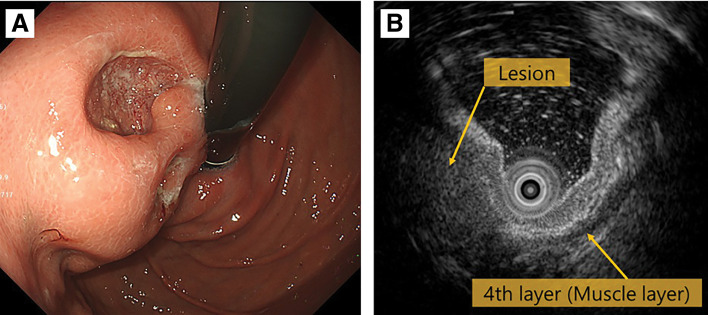
The upper gastrointestinal examination and the endoscopic ultrasound examination images. (**A**) There was a submucosal tumor with an ulcer just below the gastric cardia. (**B**) Endoscopic ultrasound revealed a hypoechoic mass with irregular margins just below the cardia, suggesting a tumor of muscularis.

**Fig. 2:**
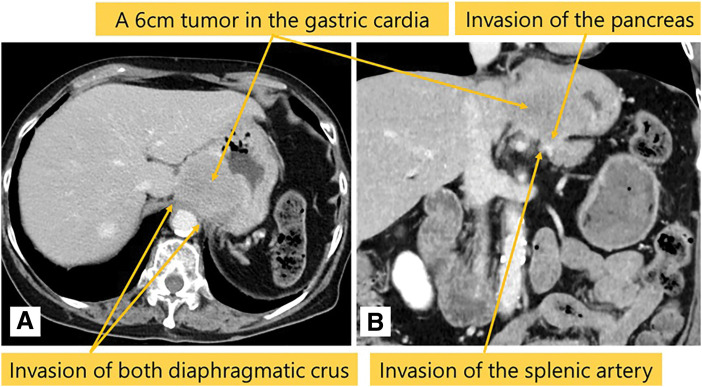
Contrast enhanced CT images. (**A**, **B**) Contrast enhanced CT revealed a 6-cm tumor in the gastric cardia with suspected invasion of the diaphragmatic crus, pancreas, and splenic artery. No other potential primary lesions were identified.

**Fig. 3:**
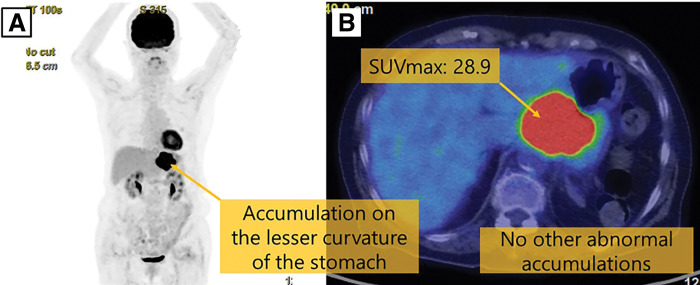
PET-CT scan images. (**A**, **B**) PET-CT scan revealed accumulation of SUV_max_ 28.9 in the tumor, but no other abnormal accumulation was noted.

**Fig. 4:**
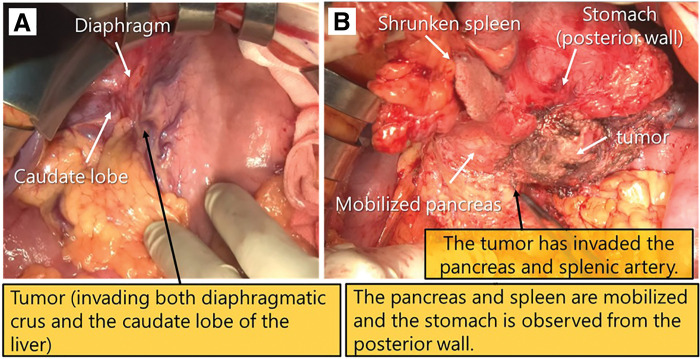
Operation findings. (**A**, **B**) A proximal gastrectomy, partial hepatectomy, distal pancreatectomy with spleen, bilateral diaphragm resection, and D2 lymph node dissection were performed.

**Fig. 5:**
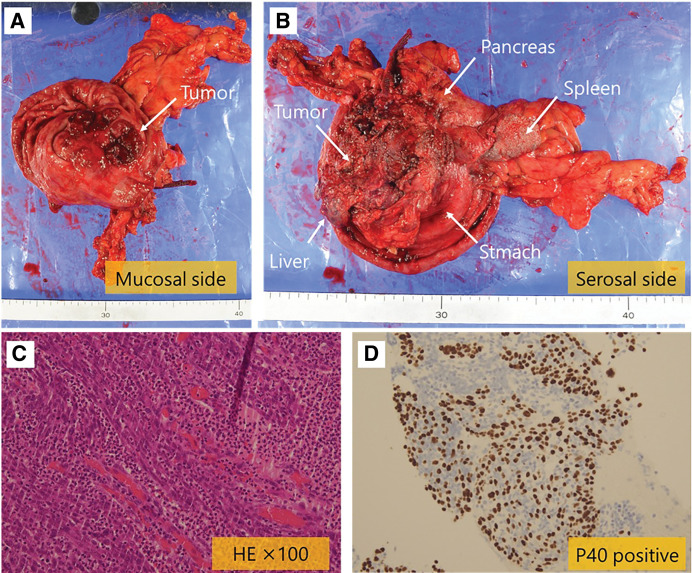
Histopathological findings. (**A**) Mucosal side of freshly resected specimen. (**B**) Serosal side of freshly resected specimen. (**C**) Hematoxylin eosin stained sections of resected tumor. (**D**) Immunohistochemistry for P40 marker of resected tumor.

The patient was a 75-year-old woman who visited a local hospital complaining of a feeling of fullness in the stomach. CT revealed a gastric submucosal tumor, and she was referred to our department. Blood tests revealed normal tumor markers (CEA, CA19-9). Upper gastrointestinal endoscopy revealed a submucosal tumor with an ulcer just below the gastric cardia. Endoscopic ultrasound revealed a hypoechoic mass with irregular margins just below the cardia, suggesting a tumor of muscularis (**Fig. 1**). Endoscopic ultrasound-guided fine needle aspiration revealed positive results for p40, GATA3, and AE1/AE3 immunoassays and an image of squamous epithelium with atypia, but it was difficult to determine the histological type or primary lesion (Group 4). Contrast-enhanced CT revealed a 6-cm tumor in the gastric cardia. The tumor showed uniform intra-tumoral contrast enhancement, and internal degeneration was unclear. The borders between the tumor and the diaphragmatic crus, pancreatic body and tail, and splenic artery were unclear, suggesting tumor invasion. No other potential primary lesions were identified. There was no significant lymphadenopathy (**Fig. 2**). PET-CT revealed that the tumor had an abnormal uptake of F-18 fluorodeoxyglucose (SUV_max_ of 28.9), but no other abnormal uptake was noted (**Fig. 3**). Based on the findings, the differential diagnosis included gastric submucosal tumor (gastric gastrointestinal stromal tumor), carcinosarcoma, gastric cancer, and malignant lymphoma. An open proximal gastrectomy, D2 lymph node dissection, partial hepatectomy, distal pancreatectomy with splenectomy, and partial resection of the diaphragm were performed (**Fig. 4**). We chose a proximal gastrectomy rather than a total gastrectomy because, even if the patient had gastric cancer, we believed that a cure could be guaranteed; as the remaining stomach was of sufficient size, the resection margins were sufficiently far apart, there were no findings suggestive of lymph node metastasis in the pyloric region, and lymph node dissection was deemed appropriate. Although a Grade II pancreatic fistula occurred, it improved with conservative treatment, and the patient was discharged 15 days after surgery. Histopathological examination showed that the tumor was surrounded by a band of lymphocytes present within the lymph nodes. Therefore, the tumor was primarily located outside the gastric wall, but involved the gastric wall and the pancreas, and protruded into the gastric mucosa. Positive p40, GATA3, and AE1/AE3 immunoassays confirmed a diagnosis of squamous cell carcinoma (**Fig. 5**). A total of 35 gastric regional lymph nodes were dissected, of which 5 showed metastatic tumor cells. All of the metastatic lymph nodes were No.3a located near the lesion. Primary squamous cell carcinoma of the stomach and pancreas was ruled out. Since there were no detectable lesions in the head and neck, esophagus, or lungs that could have been squamous cell carcinomas, the primary lesion remained unknown, and the patient was diagnosed with metastases in gastric regional lymph nodes from a CUP that had protruded into the stomach wall. The patient was administered nivolumab after surgery. There was no sign of tumor recurrence at 7 months after surgery.

## DISCUSSION

Metastases involving intraperitoneal lymph nodes from a CUP is rare and is generally classified as a poor prognostic sign.^5)^ A metastasis in a gastric regional lymph node, in particular, has been rarely reported and is extremely rare. It has been suggested that in patients with a CUP, the primary tumor may have disappeared because of the patient’s immune response.^6)^ After resection of the metastatic lymph nodes in our patient, the primary tumor remained undetectable, and she has remained free of recurrence over a short postoperative observation period of 7 months. To our best knowledge, this is the first report that has pointed out the possibility of a good prognosis if a primary tumor remains undetectable after resection of gastric regional lymph nodes containing metastatic cancer cells. This is based on the complete resection of regional lymph nodes and the hypothesis that the primary tumor disappears because of the host’s immune response. Naturally, CUP is a rare disease, so it is necessary to accumulate more cases in the future.

A CUP is a malignant disease in which a histologically confirmed metastatic lesion is identified, while the primary site remains unknown despite thorough evaluation.^1)^ A CUP is rare, accounting for only 2%–4% of all cancers.^2)^ Squamous cell carcinoma accounts for only 5%.^3)^ Because a treatment plan cannot be determined based on a specific organ, the prognosis is generally poor, with a median survival time of only 6–9 months.^7)^

A CUP can metastasize to lymph nodes as well as to the intraperitoneal organs, bones, and the brain among other sites.^1)^ Of these, an intraperitoneal metastasis has often been discovered as a metastasis to the liver or to the lymph nodes,^4)^ but is rarely discovered in the form of a gastric submucosal tumor. Only 4 cases, including this one, have been reported^8–10)^ (**Table 1**). The 3 cases involved a metastasis to the stomach, but to our knowledge, this is the first case in which a metastasis to a regional gastric lymph node enlarged sufficiently to compress the gastric wall and mimic a submucosal tumor.

**Table 1:** Reported case of cancer of unknown primary site diagnosed as a gastric submucosal tumor

Case	Author	Year	Age	Gender	Histology	Metastatic organs	Surgery	Chemotherapy	Outcome
1	Glick	1985	69	M	SCC	Stomach	No	No	Unknown
2	Mori	2016	70	F	SCC	Stomach	PG	Carbo+Doc 4 cycle	Dead 16 months
3	Tanaka	2021	67	M	Por	Stomach	DG	No	Alive 24 months
4	This case	2026	75	F	SCC	Gastric regional LN	PG	Nivolumab	Alive 7 months

Carbo, carboplatin; DG, distal gastrectomy; Doc, docetaxel; LN, lymph node; PG, proximal gastrectomy; Por, poorly differentiated adenocarcinoma; SCC, squamous cell carcinoma

Metastases to lymph nodes from CUP account for approximately 10%–40% of all cases, and it has been reported that patients with metastases limited to the lymph nodes have a favorable prognosis.^5)^ Of these, the incidence of metastases to the intraperitoneal lymph nodes is low at 4.5%. Metastases to gastric regional lymph node are extremely rare, with only 9 reported cases to date, including our case^11–18)^ (**Table 2**). In addition, the prognosis of patients with intraperitoneal lymph nodes is generally poor regardless of histological type.^1,5)^ However, all cases of gastric regional lymph node metastasis in this study had a favorable prognosis. In all cases, the primary tumor did not become apparent or was not identified, but all metastatic lymph nodes were resected. There are several hypotheses regarding the mechanism of the development of a CUP, including: 1) anatomical difficulty in detecting the primary tumor; 2) metastasis occurs before the primary tumor becomes clinically detectable; and 3) the primary tumor disappears because of the host’s immune response.^6)^ Furthermore, the frequency of a subsequent identification of the primary tumor in CUP is low. In fact, in 15%–45% of cases, the primary tumor remains unidentified, even at autopsy, and even when identified, the majority of primary tumors are smaller than 2 cm.^19,20)^ There is no evidence that re-examination of the primary tumor improves patient outcome.^21–24)^ In the 9 cases in this study, despite a long follow-up period ranging from 6 months to 10 years, the primary tumor did not become apparent or identified in any of the cases. These results suggest that the primary tumor disappears because of the host’s immune response, and the surgical resection of metastatic lymph nodes may achieve an outcome equivalent to an R0 resection.

**Table 2:** Reported case of gastric lymph node metastasis from cancer of unknown primary site

Case	Author	Year	Age	Gender	Preoperative diagnosis	Surgery	Histology	Chemotherapy	Recurrence	Primary site	Outcome
1	Nishimura	1995	53	M	Intraperitoneal tumors	Intraperitoneal tumor resection	Por	5FU+MMC	No	Unidentifiable	Alive 10 years
2	Kimura	2005	85	F	Pancreatic head tumor	PD	Adeno	No	No	Unidentifiable	Alive 8 years
3	Kusumoto	2011	54	M	No.8 LN tumor	Lymphadenectomy	SCC	FP→S-1→DOC	LN	Unidentifiable	Alive 4 years
4	Lee	2012	52	M	Gastric cancer	STG	NEC	Etoposide+CDDP	No	Unidentifiable	Unknown
5	Ito	2014	76	F	No.3a, 8 LN tumor	Lymphadenectomy	Adeno	No	No	Unidentifiable	Alive 5 years
6	Seshie	2020	65	M	Gastric cancer	DG	Adeno SCC	No	No	Unidentifiable	Alive 2 years
7	Nakamura	2021	60	F	Gastric cardia LN metastasis of CUP	No.7,9,11p LN tumor resection	SCC	S-1	No	Unidentifiable	Alive 2.5 years
8	Morimoto	2024	68	M	No.8 LN metastasis of CUP	No.5, 8a LN tumor resection	Por	No	No	Unidentifiable	Alive 6 months
9	This case	2026	75	F	Gastric submucosal tumor	PG	SCC	Nivolumab	No	Unidentifiable	Alive 7 months

5FU, 5-fluorouracil; Adeno, adenocarcinoma; CDDP, cisplatin; CUP, cancer of unknown primary site; DG, distal gastrectomy; DOC, docetaxel; FP, 5-fluorouracil and cisplatin; LN, lymph node; MMC, mitomycin C; NEC, neuroendocrine carcinoma; PD, pancreaticoduodenectomy; PG, proximal gastrectomy; Por, poorly differentiated adenocarcinoma; S-1, tegafur/gimeracil/oteracil potassium; SCC, squamous cell carcinoma; STG, subtotal gastrectomy

Advances in immune checkpoint inhibitor therapy have been attracting attention in regard to the treatment of patients with CUP. Following the results of the NivoCUP trial,^4)^ nivolumab was approved in Japan as the first anticancer drug for CUP. The response rates in 19 patients with metastatic cancer limited to the lymph nodes and 37 patients with other metastases were 36.8% and 13.5%, respectively, and the 6-month progression-free survival rates were 41% and 25%, respectively. Nivolumab demonstrated a higher efficacy in patients with metastatic cancer in the lymph nodes only. While the precise mechanism remains unclear, the immunological background suggested that nivolumab may be effective.^25)^ This case also showed metastatic cancer in the lymph nodes only, and we hope that nivolumab will be effective.

## CONCLUSIONS

We successfully treated a rare case of CUP presenting as lymph node–only metastasis to the regional gastric lymph nodes with an R0 resection. The patient remained recurrence-free postoperatively, suggesting that complete resection of the regional lymph nodes may provide a favorable prognosis in selected patients with CUP and lymph node–only metastasis. Naturally, CUP is a rare disease, so it is necessary to accumulate more cases in the future.

## DECLARATIONS

### Availability of data and materials

Data sharing is not applicable to this article, as no datasets were generated or analyzed.
